# Capturing virus evolution by proteomic bioinformatics: Hunting for characteristic mutations in the hepatitis E virus genome

**DOI:** 10.1080/21505594.2017.1384526

**Published:** 2017-11-10

**Authors:** Dominik Harms, Bo Wang, C. Patrick Papp, C.-Thomas Bock

**Affiliations:** a Division of Viral Gastroenteritis, Hepatitis Pathogens and Enteroviruses, Department of Infectious Diseases, Robert Koch Institute, Berlin, Germany; b Institute of Tropical Medicine, University of Tuebingen, Tuebingen, Germany

**Keywords:** bioinformatics, epitopes, evolution, genotype, hepatitis e virus, mutation, virus diversity

In the recent issue of *Virulence*, Ikram et al. have compiled an extensive proteomic bioinformatics study of characteristic mutations in the hepatitis E virus (HEV) genome. This study showed genotype-specific prevalence of respective mutations and demonstrated the selective pressure imposed upon the virus by both host immune defense and antivirals.^1^

The evolutionary significance of virus infections has been a subject of discussion for decades;^2^ however, modern *omics*-analyses (e.g., proteomics, transcriptomics) and bioinformatics became crucial when it became apparent that genetic variants of viruses were associated to pathogenesis and disease progression. The first article in terms of virus diversity and course of disease was published by Pierre Lépine in 1938 who reported on the evolution of different rabies virus strains and their association to different characteristics of infectivity and virulence.^3^ More recent reports have demonstrated the significance of viral evolution in the pathogenesis of virus infections, in particular of RNA viruses (HCV, HEV, influenza virus) and HIV infections.^4–8^

Long thought to be a healthcare burden exclusive to developing countries with sporadic cases in industrialized countries confined to travel-borne infections, HEV is now being increasingly recognized as an emerging disease globally.^9^ Reports of autochthonous cases of HEV infections with zoonotically transmitted HEV genotypes 3 and 4 have drastically increased in industrialized countries in the last years.^10,11^ Although most cases remain asymptomatic or present as acute self-limiting viral hepatitis, serious disease outcomes may result in certain at-risk populations. Hepatic failure and high mortality rates are common in HEV patients with preexisting liver disease as well as infected pregnant women.^12,13^ Chronic infections related to HEV genotypes 3 and 4 can occur in immunocompromised persons such as organ transplant recipients and HIV positive patients.^14,15^ Beyond this, HEV may cause a wide spectrum of extrahepatic manifestations.^16^ Off-label administration of ribavirin monotherapy have shown to be effective in cases of chronic infection, but their use has also laid bare the ongoing struggle between the human immune system, antiviral treatment, and HEV evolution.^17,18^

Viral adaptation as a response to host innate and adaptive immune response and selective pressure by antiviral treatment is critical to ensure continued viral fitness and replication competency. Studies on chronic infections with hepatitis B virus (HBV) and C virus (HCV) are prime examples of how viral evolution can overcome host immune defenses and antiviral therapy. For example numerous immune-driven adaptations within nonstructural regions of HCV have been described, including genotype-specific HLA-associated escape mutations.^19,20^ In addition, low efficacy of interferon-based and nucleoside analogue therapy due to viral heterogeneity and development of drug-resistant viral quasispecies has been a common issue in treatment of chronic HBV and HCV patients.^21–23^ These circumstances have long since underlined the necessity to better understand the mechanisms involved in viral replication, the functions of adaptive mutations, and to ultimately develop more efficient treatment options, including patient-tailored protocols and direct-acting antivirals.^24^

Unfortunately, we are far from having reached this goal in the control of HEV. As a single-strand positive-sense RNA virus, HEV genome replication is extremely error-prone since HEV RNA-dependent RNA polymerase lacks proofreading activity and thus generates mutations rapidly during transcription, undoubtedly contributing to viral evolution. A plethora of mutations within the three HEV open reading frames (ORFs) have been described *in vitro* and *in situ*.^8^ However, those described in patient-specific isolates may have the most important impact on clinical application. Changes in the HEV proteome are diverse and not well understood. Several amino acid changes have been associated with hepatic failure (e.g., F179S, C1483W, N1530T) and antiviral ribavirin therapy failure (G1634R/K) while larger insertions into the hypervariable region (HVR) often appear in the context of chronic hepatitis E.^25–28^

In light of the accumulating descriptions of patient-specific HEV proteome alterations, there is an urgent need to analyze their global prevalence and assess their association to clinical outcomes. Undoubtedly, the optimal antiviral treatment ensuring sustained virological response is a personalized treatment strategy that targets individual host and viral factors. Individualized therapy could be optimized if treatment-naive viral quasispecies and functional mutations have been identified and assessed before treatment starts. This in turn requires a comprehensive analysis of the true prevalence and significance of relevant mutations described so far.

In their article under discussion, Ikram and colleagues employed elegant large-scale proteomic bioinformatics to shed new light on previously described HEV mutations associated with ribavirin treatment failure, chronic infection, hepatic failure and altered immunoreactivity. They carefully assessed the global prevalence of characteristic mutations among different HEV genotypes (HEV-1, HEV-3, and HEV-4), their impact on B an T cell epitope reactivity and their genotype-specific evolution.^1^ The question posed by Ikram *et al.* at the onset of their study was to identify which effects specific HEV variants can have on susceptibility, pathogenesis of infection and treatment outcome.^1^ After exploring the GenBank and literature for clinical and *in vitro* data for mutations in the HEV ORF1 and ORF2 regions the investigators identified 20 characteristic amino acid variants (17 in ORF1 and 3 in ORF2). In a next step they assessed the prevalence of the 20 mutations within 406 full-length ORF1 and ORF2 sequences of different HEV genotypes, while taking into account their association with hepatic failure, treatment failure, chronicity and altered immunoreactivity. The authors made the astounding realization that specific mutations (9 out of 17 in ORF1, and 1 of 3 in ORF2) were conserved and by contrast the remaining analyzed variations showed a high degree of variability. The latter includes two notable mutations T735I and G1634R/K that were frequent among the major human pathogenic HEV genotypes 1, 3, and 4 and correlated with liver failure and ribavirin therapy failure, respectively. Furthermore, two amino acid residues Leu477 and Leu613 within the capsid protein (ORF2) known to be involved in formation of neutralization-sensitive epitopes were mutated with considerably high frequency in HEV genotype 1 and 3 implicating that immune evasion is a common strategy in HEV infection.

Next, the investigators utilized *in silico* SNP algorithms to predict whether the investigated mutations were deleterious and caused protein destabilization, respectively. Their finding indicates that most ORF1 mutations and 1 of 3 ORF2 mutations are indeed deleterious and that notably two ORF2 substitutions (P259S, L477T) can destabilize the capsid protein structure.

The authors went on to investigate whether the described mutations resulted in altered B and T cell epitope antigenicity based on the hypothesis that acquisition of escape mutations may hide the virus from host defenses. Through a combination of epitope prediction programs and online databases B and T cell epitopes (class MHCI and MHCII) of HEV genotype 1, 3 and 4 consensus sequences were explored. Intriguingly, overlap of epitopes and mutation sites were found for many of the studied substitutions. Subsequent evaluation revealed reduced antigenicity of mutated epitopes as compared to wild-type with only few having retained or increased antigenicity. These findings strongly imply that, similarly to HBV and HCV, HEV may profit from altered epitopes via decreased B and T cell recognition.

Finally, Ikram and colleagues mapped the evolution of characteristic mutations for each HEV genotype. Notably, the investigators found that especially the mutations T735I, the ribavirin failure-associated G1643R/K and leucine substitutions L477T and L613T in ORF2 developed earlier in HEV genotype 1 than 3 and thus evolution of HEV-1 may have occurred earlier than HEV-3 and -4.

Taken together, Ikram and colleagues have addressed an important issue of virus infection, namely virus evolution and the relevance of characteristic mutations in the viral genome. The in-depth approach using large-scale proteomic bioinformatics to characterize the intra-genotypic prevalence and evolution of HEV variants and their implications for clinical outcomes and host immune evasion is a prime example of how bioinformatics can be a valuable tool to better understand the functions of viral adaptation. As shown also for other viruses (e.g., HBV, HCV, HIV, HCMV), the course of antiviral treatment can be adjusted and optimized if host and viral factors are assessed for each individual patient. The high mutation rate of HEV, the frequent emergence of ribavirin failure-associated quasispecies and the ability of HEV to hide from host immune defense are factors that underline the necessity to profile and study characteristic viral adaptations. There is therefore need to invest in modern techniques since the powerful next generation sequencing approaches (in-depth, whole genome etc.) and digital epidemiology including intensified molecular surveillance and proteomic bioinformatics will provide us with the necessary tools to identify and discriminate characteristic and relevant variations from polymorphisms in the viral genome. More detailed knowledge about virus evolution and diversity will help us not only to understand the pathophysiology of virus infection and virus-host interactions but also to better manage infected patients. Accordingly, the findings from Ikram and colleagues add new insights to our knowledge of the complex processes of viral evolution, viral diversity, and selection of HEV quasispecies. We are still at the early stages of characterizing viral diversity and its association with pathogenesis, immune defense, and therapy efficacy. The paper under discussion is an excellent example of how to explore virus diversity and its impact on the course of infection using sophisticated methods. Only then can the burden of serious hepatitis E be lifted.
